# Recent Issues in the Development and Application of Targeted Therapies with Respect to Individual Animal Variability

**DOI:** 10.3390/ani15030444

**Published:** 2025-02-06

**Authors:** Natalia Kurhaluk, Halina Tkaczenko

**Affiliations:** Institute of Biology, Pomeranian University in Słupsk, Arciszewski St. 22b, 76-200 Słupsk, Poland; halina.tkaczenko@upsl.edu.pl

**Keywords:** species-specific treatment strategies, signalling pathways, epigenetic and paragenetic influences, free radicals and oxidative stress

## Abstract

This literature review explores the impact of molecular, genetic, and environmental factors on the efficacy of targeted therapies in veterinary medicine. Relevant studies were identified through systematic searches of PubMed, Web of Science, Scopus, and ScienceDirect using keywords such as “species-specific treatment strategies”, “signalling pathways”, “epigenetic and paragenetic influences”, “targeted therapies”, “veterinary medicine”, “genetic variation”, and “free radicals and oxidative stress”. Inclusion criteria included studies focusing on species-specific therapeutic responses, genetic influences, and oxidative stress. To ensure that only the most recent and relevant evidence was included, only peer-reviewed publications from the last two decades were considered. Each study selected for analysis was critically appraised, with a particular emphasis on methodological quality, experimental design, and scientific contribution to the understanding of how environmental and biological factors influence therapeutic outcomes. A special emphasis was placed on studies that used a comparative, cross-species approach to assess variability in therapeutic responses and potential adverse effects. The review synthesises evidence on the role of epigenetic and paragenetic factors and highlights the importance of cross-species studies to understand how environmental and biological factors influence treatment outcomes. By highlighting genetic variation, oxidative stress, and individual species differences, the review argues for personalised and species-specific therapeutic approaches. The review emphasises that such an approach would improve veterinary care and inform future research aimed at optimising targeted therapies, ultimately leading to better animal health and treatment efficacy. A key contribution of the review is its emphasis on the need for more personalised treatment protocols that take into account individual genetic profiles and environmental factors; it also calls for a greater integration of cross-species studies.

## Simple Summary

This study reviews targeted therapies in veterinary medicine, focusing on how specific treatments can improve animal care by addressing the underlying causes of disease. The research highlights the role of genetics, environment, and other factors that influence the effectiveness of treatments in different species, with the main aim of exploring how individual differences—such as genetic and environmental factors—affect an animal’s response to therapies. By understanding these variations, veterinarians can formulate individualised treatment plans to ensure optimal outcomes. The study highlights the importance of comparing the responses of different species to therapies, helping to identify potential risks and benefits. This knowledge will help to refine treatment strategies and ensure the safety and efficacy of therapies for different animal populations. The results highlight the need for further research to improve our understanding of the interplay between biology and environment in determining therapeutic success. The knowledge gained from this research could contribute to the advancement of veterinary medicine, the promotion of animal health, and the development of new opportunities for the creation of more targeted therapies that benefit both animals and humans, thus improving overall healthcare.

## 1. Introduction

The importance of targeted therapies in veterinary medicine cannot be overstated, as they represent a significant advance in the way diseases are diagnosed and treated in animals. Unlike traditional therapies, which often take a one-size-fits-all approach, targeted therapies focus on specific molecular pathways and genetic factors that contribute to disease processes. This precision allows for more effective and tailored treatment options, ultimately leading to improved health outcomes in different species, and has been confirmed by research [1,2]. In the context of veterinary medicine, the ability to understand targeted therapies is of paramount importance in optimising patient care and ensuring that the most appropriate treatment is delivered to each individual animal based on its unique biological characteristics. The complexity of animal diseases requires a comprehensive analysis of the underlying mechanisms that drive their pathology. By studying the molecular and genetic basis of disease, veterinarians can improve their understanding of how different species respond to therapies, including the influence of genetic variation, immune responses, and environmental factors. This knowledge is essential not only for the development of new treatment protocols, but also for predicting treatment efficacy and potential adverse effects. As a result, a broad understanding of targeted therapies is essential to advance veterinary practice and ensure that animals receive safe and effective care, as has been demonstrated in several studies [2,3].

Many diseases that affect animals are also prevalent in humans; the study of targeted therapies in veterinary contexts can provide valuable information for the development of treatments for similar conditions in humans, as has been demonstrated in various studies [4,5]. This interrelationship highlights the importance of rigorous cross-species research and collaboration, as advances in one field can have a significant impact on the other. Therefore, a thorough investigation of targeted therapies in veterinary medicine is not only relevant to animal health, but also has broader implications for translational medicine and the overall advancement of healthcare [6].

## 2. Methodology

A systematic search of major academic databases, including PubMed, Web of Science, Scopus and ScienceDirect, was conducted to identify relevant studies. The selection criteria emphasised peer-reviewed articles published within the last two decades, with a priority given to studies addressing genetic, epigenetic and paragenetic influences on treatment outcomes. The included studies were required to demonstrate relevance to species-specific treatment responses, the role of oxidative stress, and the impact of genetic variation on therapeutic efficacy. Exclusion criteria included studies that lacked methodological rigour or relevance to targeted therapies in veterinary contexts. A comprehensive search was conducted using specific keywords, such as ‘targeted therapies’, ‘veterinary medicine’, ‘genetic variation’, ‘oxidative stress’ and ‘species-specific treatment’, to collect data from different sources. Each study was critically appraised for methodological quality, experimental design, and scientific contribution to the understanding of how environmental and biological factors influence therapeutic outcomes.

The significance and originality of this review lies in its emphasis on personalised, species-specific targeted therapies in veterinary medicine. In contrast to many existing studies, it highlights the role of genetic, epigenetic, and environmental factors in shaping therapeutic responses in different species. By integrating these diverse elements, the review provides a novel perspective on optimising treatment efficacy and minimising risks. Its emphasis on cross-species comparative studies and individualised care provides important insights that can improve therapeutic protocols and animal health outcomes in veterinary practice.

This study aims to investigate the molecular mechanisms of targeted therapies in animals, focusing on key pathways and molecular targets to develop more precise therapeutic strategies. A key aim is to investigate the influence of genetic factors on the variable responses to these therapies in different species, with a particular focus on how gene expression affects treatment outcomes. Despite significant advances in the field, there remains a gap in the understanding of how genetic variability and gene expression influence therapeutic efficacy in veterinary medicine. Filling these knowledge gaps is essential for the development of personalised treatment protocols that minimise adverse effects and enhance therapeutic efficacy. Recognising the central role of genetics in treatment response has the potential to optimise therapeutic interventions and thereby improve outcomes for a range of animal diseases [7,8].

### 2.1. Molecular and Genetic Basis of Targeted Therapies in Animals

**Figure 1:**
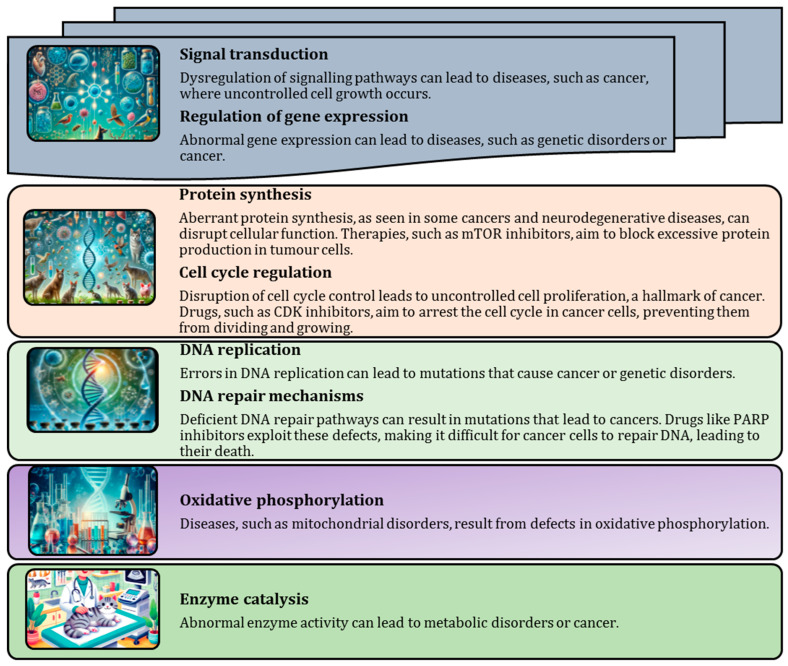
Molecular processes are fundamental to the development of disease and the efficacy of therapies, as they influence cellular functions, gene expression, and the regulation of biological pathways. Understanding these processes is crucial for the development of targeted therapies; molecular changes during disease progression often determine the success or failure of treatments and thus guide personalised healthcare strategies [11,12,13,14,15,16,17,18,19,20,21,22,23,24,25,26,27,28,29,30,31,32,33,34,35,36,37,38,39].

Molecular processes are intricately linked to disease and therapy, as dysfunction in pathways, such as signalling, transcription, DNA replication and apoptosis, can lead to diseases such as cancer, genetic disorders, and metabolic diseases [9,10,11,12]. Targeted therapies aim to correct these dysfunctions by inhibiting specific enzymes, restoring cell cycle control, or promoting proper apoptosis, ultimately improving treatment outcomes and precision, as shown in Figure 1.

These therapies often aim to inhibit or activate specific signalling pathways within cells, such as those that regulate cell growth, immune response, or apoptosis (programmed cell death), as shown by various authors [11,12,13]. Advances in molecular biology have enabled scientists to map these pathways in detail, leading to the development of drugs that precisely target abnormal proteins or pathways, thereby reducing damage to healthy cells [13].

Targeted therapies have been extensively studied and developed for a wide range of animal diseases, with a particular focus on cancer, immune disorders, infectious diseases, and other chronic conditions. The basic mechanism of these therapies is to selectively target specific molecular pathways or biomarkers that are dysregulated in diseased tissues. In the field of oncology, targeted therapies have been used to treat various cancers, including canine lymphoma, by inhibiting the expression of specific proteins involved in cell proliferation and survival such as tyrosine kinases [14,15]. In canine mast cell tumours, targeted drugs focus on blocking the activity of specific growth factor receptors, such as the KIT receptor, which is often mutated and drives tumourigenesis [16,17]. In the context of feline mammary carcinoma, molecular therapies are designed to target oestrogen receptor pathways and inhibit tumour cell growth [18,19]. Similarly, in equine melanoma, treatments aim to modulate immune responses or target specific melanoma-associated antigens [20,21].

In addition to their use in the treatment of oncological diseases, targeted therapies have demonstrated efficacy in the treatment of autoimmune and inflammatory diseases. For example, in the context of canine atopic dermatitis, therapies targeting immune system mediators, including interleukins or tumour necrosis factor (TNF), aim to reduce inflammation and allergic responses [22,23]. In rheumatoid arthritis, targeted therapies are designed to inhibit pro-inflammatory cytokines, such as TNF-α, that play a key role in joint destruction and subsequent inflammation [24]. Another condition where targeted therapies are being explored is canine pulmonary hypertension, where the focus is on modulating vascular endothelial growth factor (VEGF) and other pathways involved in vascular remodelling and vasoconstriction [25].

Targeted approaches have also been used in infectious diseases, as exemplified by the development of therapies designed to inhibit the replication of the virus causing feline infectious peritonitis (FIP). These therapies target the enzymatic machinery of the virus, specifically the proteases and polymerases involved in viral replication [26,27]. A similar approach is used in the treatment of canine leishmaniasis, where therapies focus on targeting the parasite’s ability to evade immune responses and inhibiting its metabolic pathways to reduce the parasite burden and improve host response [28].

In endocrine disorders, such as canine hyperadrenocorticism (Cushing’s disease), therapies target the adrenal glands and pituitary–adrenal axis to reduce excessive cortisol production by inhibiting key enzymes such as 11β-hydroxylase [29,30]. In the case of insulinoma, targeted therapies aim to regulate insulin production by modulating specific signalling pathways that control insulin secretion in pancreatic β-cells in both ferrets and dogs [31].

Therapeutic interventions for neurological diseases, such as canine epilepsy, focus on altering neurotransmitter pathways, including GABA and glutamate, to restore normal neuronal activity [32]. In equine protozoal myeloencephalitis (EPM), treatments aim to inhibit the invasion of neural tissue by the protozoan parasite by targeting the host’s immune response and disrupting its metabolism [33]. Chronic kidney disease in cats also benefits from targeted therapies that focus on regulating the inflammatory mediators and oxidative stress pathways involved in renal damage [33]. In addition, therapeutic interventions for canine progressive retinal atrophy (PRA) target the modulation of visual signalling pathways and the protection of retinal cells from oxidative damage [35,36,37,38]. In the context of osteoarthritis, targeted therapies are being developed to modulate the inflammatory cytokines and enzymes responsible for cartilage degradation in dogs and horses [35,36,37,38].

Signalling pathways are essential for understanding the mechanisms underlying various animal diseases and may serve as potential therapeutic targets for more effective treatments [39]. A considerable body of evidence has pointed to the fact that the Janus kinase (JAK) signalling pathway mediates cytokine signalling and is involved in autoimmune diseases, such as canine atopic dermatitis and rheumatoid arthritis [40,41], while the TNF-α signalling pathway is critical in the inflammatory responses associated with these diseases, as was demonstrated in some publications [42,43].

Numerous studies have established that the phosphoinositide 3-kinase (PI3K)/Akt and mitogen-activated protein kinase (MAPK) pathways are often dysregulated in cancers, such as canine lymphoma and mast cell tumours [44,45,46,47], affecting cell growth and survival. The vascular endothelial growth factor (VEGF) signalling pathway is essential for angiogenesis and is targeted in diseases, such as canine osteosarcoma, to inhibit tumour blood supply [48,49]. The nuclear factor kappa B (NF-kB) signalling pathway regulates immune responses in autoimmune diseases [50].

**Figure 2:**
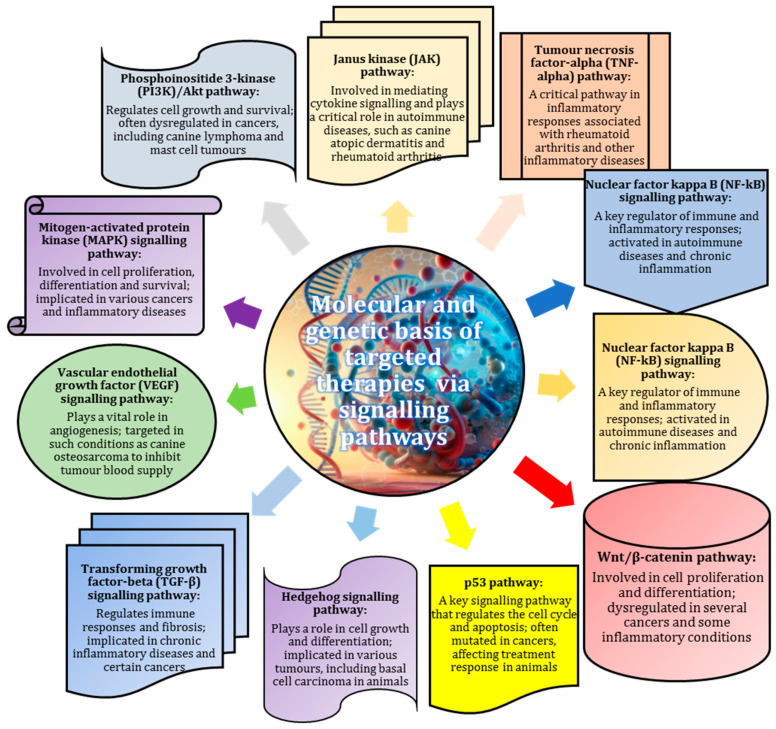
Key molecular and genetic components involved in targeted signalling therapies. The figure highlights the specific pathways, receptors, enzymes, and genetic alterations that play a critical role in the regulation of cellular processes. These elements are essential for understanding how targeted therapies can selectively modulate disease mechanisms to improve treatment precision and outcomes [40,41,42,43,44,45,46,47,48,49,50,51,52,53,54].

A body of research underlines the significance of other important signalling pathways, including, as follows: Wnt/β-catenin (involved in cancer) [51]; transforming growth factor-beta (TGF-β) (associated with chronic inflammation) [52]; the p53 signalling pathway (involved in tumour apoptosis) [53]; and the Hedgehog signalling pathway (involved in various tumours) [54]. Understanding these pathways is essential for the development of targeted therapies that can effectively manage and treat these diseases in animals (Figure 2).

#### 2.1.1. Cell Growth

A synthesis of current research illustrates how cell growth is tightly regulated by several signalling pathways that ensure proper development, tissue maintenance, and repair. One of the most important signalling pathways involved in cell growth is the PI3K/AKT/mTOR pathway, which controls cell proliferation, growth, and survival, as shown in research experiments [55]. This pathway is activated by growth factors that bind to receptor tyrosine kinases (RTKs) on the cell surface. Upon activation, PI3K (phosphoinositide 3-kinase) catalyses the production of PIP3, which recruits AKT (protein kinase B) to the membrane. AKT then activates mTOR (mechanistic target of rapamycin), a key regulator of cell growth and protein synthesis, as determined by various research teams [56]. Research conducted by Glaviano et al. (2023) showed that dysregulation of this pathway, often through mutations or overactivation, leads to uncontrolled cell growth, as seen in several cancers [55]. Furthermore, it has been shown that targeted therapies that inhibit the key components of the PI3K/AKT/mTOR pathway can effectively retard or stop cancer cell proliferation [57].

#### 2.1.2. Immune Response

Evidence presented by Chaplin (2010) indicates that the immune response is regulated by complex signalling networks that control the activation, proliferation, and differentiation of immune cells [58]. The JAK/STAT signalling pathway is a primary signalling mechanism for several cytokines and growth factors that are critical for immune regulation. When a cytokine binds to its receptor, associated Janus kinases (JAKs) are activated, leading to the phosphorylation and activation of signal transducer and activator of transcription (STAT) proteins. Activated STATs translocate to the nucleus to induce the transcription of immune-related genes. Studies [59,60] have demonstrated that this pathway is critical for processes such as inflammation, immune cell activation, and defence against pathogens. Aberrations in JAK/STAT signalling can lead to autoimmune diseases or chronic inflammation [61]. Studies have shown that targeted therapies that inhibit JAK activity (e.g., JAK inhibitors) can be used to treat autoimmune diseases, e.g., rheumatoid arthritis [62,63].

#### 2.1.3. Apoptosis and the Intrinsic/Extrinsic Pathways

A body of work in this area has consistently demonstrated that apoptosis, or programmed cell death, is a vital process that eliminates damaged or unwanted cells. It is regulated by two main signalling pathways, the intrinsic (mitochondrial) and extrinsic (death receptor) pathways [64]. The intrinsic pathway is triggered by internal stress signals, such as DNA damage or oxidative stress. This leads to the release of cytochrome c from the mitochondria, which activates caspase-9, initiating a cascade of caspase enzymes leading to cell death. The extrinsic pathway is activated by the binding of death ligands (e.g., FasL or TNF) to death receptors on the cell surface, triggering caspase-8 activation and subsequent cell death. Apoptosis is tightly regulated by proteins Bcl-2 (which inhibits apoptosis) and Bax (which promotes apoptosis) [65,66]. A considerable body of evidence has pointed to the fact that dysregulation of apoptotic pathways can lead to diseases, such as cancer, where cells evade apoptosis, or to neurodegenerative diseases, where excessive apoptosis occurs [67]. The findings of Carneiro and El-Deiry (2020) confirm that therapies targeting these pathways aim to restore the balance by either promoting apoptosis in cancer cells or inhibiting it in diseases with excessive cell death [68].

#### 2.1.4. Cell Proliferation and Differentiation

The results of studies have corroborated the hypothesis that the MAPK/ERK (mitogen-activated protein kinase/extracellular signal-regulated kinase) signalling pathway is a critical signalling mechanism involved in the control of cell proliferation, differentiation, and survival. The pathway is typically activated by growth factors and involves a cascade of phosphorylation events starting with the activation of RAS, followed by the sequential activation of Raf, MEK, and ERK. Once activated, ERK translocates to the nucleus, where it regulates the expression of genes involved in cell division and differentiation [69,70]. Dysregulation of the MAPK/ERK pathway, such as mutations in the *RAS* or *RAF* genes, can lead to excessive cell division and cancer progression [71]. Earlier studies by Sanchez et al. (2018) have indicated that targeted inhibitors, such as MEK and RAF inhibitors, can be used to treat cancers, such as melanoma, where these mutations are common [72].

#### 2.1.5. Immune Regulation and Inflammation

The NF-κB (nuclear factor kappa-light-chain-enhancer of activated B cells) signalling pathway plays a central role in the regulation of immune responses, inflammation, and cell survival. According to findings by Liu et al. (2017), it is activated by a variety of stimuli, including cytokines, pathogens, and stress signals [73]. Once activated, NF-κB proteins translocate from the cytoplasm to the nucleus where they promote the expression of genes involved in inflammation, immune cell activation, and survival. This pathway is critical for defence against infection; however, research has shown that chronic activation can lead to diseases such as autoimmune disorders, chronic inflammation, and cancer [74,75]. For example, in certain cancers, the constitutive activation of NF-κB promotes tumour survival and resistance to apoptosis [76]. Findings from studies by Brown et al. (2008) and Guo et al. (2024) provide compelling evidence regarding these mechanistic pathways. Targeted therapies that inhibit NF-κB signalling are being investigated as potential treatments for inflammatory diseases and cancers with aberrant NF-κB activity [75,77].

#### 2.1.6. Cancer

A thorough examination of the existing literature reveals that the types of cancer in animals and the particular challenges associated with them are most commonly discussed in medical practice, as cancer is a major health concern in veterinary medicine, affecting various species, particularly dogs and cats. Common types include lymphoma, mast cell tumours, osteosarcoma, and mammary carcinoma. Each type presents unique biological behaviours, treatment responses, and challenges [78,79]. For example, canine lymphoma can manifest in multicentric, gastrointestinal, or cutaneous forms, complicating diagnoses and treatment strategies. Canine mast cell tumours often have variable histological grades affecting prognosis and therapeutic decisions [80]. In addition, the tendency of certain tumours, such as osteosarcoma, to metastasise poses significant challenges to treatment and management, and requires tailored therapeutic approaches [81].

It is evident from the research that canine lymphoma is a common haematological malignancy characterised by the proliferation of lymphocytes in lymphoid tissues [82]. Targeted therapies, in particular tyrosine kinase inhibitors (TKIs), have emerged as a promising approach to treatment [83]. One of the best known TKIs, toceranib, specifically inhibits signalling pathways activated by growth factor receptors. By blocking these pathways, toceranib disrupts the survival signals that promote lymphoma cell proliferation and survival [84]. Research has shown that toceranib can significantly improve outcomes in dogs with certain subtypes of lymphoma, providing a more targeted approach than conventional chemotherapy, which can be more toxic and less specific [84].

Numerous studies have established that canine mast cell tumours (MCTs) are one of the most common skin tumours in dogs and are often driven by mutations in receptor tyrosine kinases, particularly the *KIT* gene [85]. Targeted therapies, such as masitinib and toceranib, have been developed to specifically inhibit these mutated receptors. By blocking the signalling pathways activated by aberrant *KIT* mutations, these drugs can effectively reduce tumour growth and metastasis [86]. Masitinib, for example, not only inhibits KIT but also targets other tyrosine kinases involved in mast cell activation and survival, making it a multi-faceted treatment option, as shown in various experiments [87]. Clinical trials have shown that these targeted therapies can lead to significant improvements in tumour control and overall survival in dogs with MCTs [88].

A synthesis of current research illustrates how the targeting of KIT signalling in equine melanoma is an innovative approach that focuses on inhibiting the aberrant pathways driven by mutations in the *KIT* gene commonly associated with this type of cancer in horses. By using specific inhibitors or monoclonal antibodies that block KIT activation, this strategy aims to reduce tumour growth and metastasis, ultimately improving the prognosis and quality of life for affected horses [89]. This targeted therapy not only holds promise for improving treatment efficacy but also highlights the need for personalised veterinary care that takes into account the unique genetic and molecular characteristics of equine melanoma.

Various studies have shown that feline mammary carcinoma is known to be a common form of cancer in cats and is often difficult to treat due to its aggressive nature [90]. Recent research has focused on targeted therapies using specific molecular targets such as HER2 (human epidermal growth factor receptor 2) and other growth factor receptors. The overexpression of HER2 has been observed in some feline mammary tumours, similar to that observed in certain human breast cancers. Targeted therapies, such as trastuzumab, that specifically inhibit HER2 signalling are being investigated for their efficacy in the treatment of these tumours. Studies, such as one by Gameiro et al. (2021), have shown that by blocking HER2-mediated signalling pathways, these therapies could potentially reduce tumour cell proliferation and improve outcomes for affected cats, as shown in some experiments [18].

Numerous studies have established that osteosarcoma is an aggressive bone tumour that is common in dogs and often has a poor prognosis due to its tendency to metastasise [91,92,93]. Research by Makielski et al. (2019) suggests that one of the key features of tumour growth is the process of angiogenesis, i.e., the formation of new blood vessels to supply the tumour with nutrients [92]. Targeted therapies aimed at inhibiting vascular endothelial growth factor receptor (VEGFR) signalling have gained attention in the treatment of canine osteosarcoma. By using VEGFR inhibitors, researchers aim to cut off blood supply to the tumour, effectively starving the neoplasm and preventing its further growth [93].

Studies have shown that combining VEGFR inhibitors with conventional therapies can increase the overall effectiveness of treatment and improve survival rates in dogs suffering from osteosarcoma [94]. Inhibiting angiogenesis in this aggressive canine cancer is a promising therapeutic strategy that aims to disrupt the formation of the new blood vessels necessary for tumour growth and metastasis. By targeting VEGFRs, therapies can effectively reduce tumour blood supply, ultimately leading to reduced tumour size and improved survival rates for affected dogs [95]. This approach not only enhances the efficacy of traditional treatments, but also offers a more targeted method of combating this aggressive cancer, paving the way for improved outcomes for canine patients diagnosed with osteosarcoma [96].

**Figure 3:**
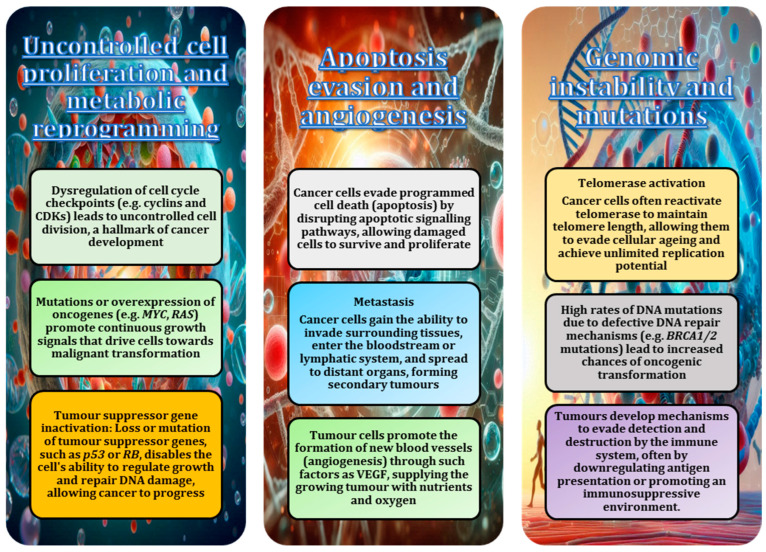
Cancer development in different animal species is driven by a number of key mechanisms, including uncontrolled cell proliferation, metabolic reprogramming, evasion of apoptosis, angiogenesis, and genomic instability. These mechanisms collectively drive tumour initiation, progression, and metastasis across species [78,79,80,81,82,83,84,85,86,87,88,89,90,91,92,93,94,95,96,97,98,99].

Recent advances in molecular biology and oncology have led to the development of targeted therapies specifically designed to treat cancer in animals. These therapies often focus on inhibiting specific pathways or molecular targets involved in tumour growth and survival, as shown in various analyses [5,97]. For example, tyrosine kinase inhibitors, such as toceranib and masitinib, have shown promise in the treatment of canine mast cell tumours by specifically targeting mutated receptor pathways [86]. In addition, immunotherapeutic strategies, such as the use of monoclonal antibodies, have emerged as effective treatments for certain cancers by enhancing the ability of the immune system to recognise and destroy cancer cells [98]. These targeted therapies offer the potential for more effective treatment with fewer side effects than traditional chemotherapy. Key mechanisms involved in the development of cancer are shown in Figure 3. These mechanisms are common to cancers in different species and highlight the complex interplay between genetic, molecular, and environmental factors in the progression of the disease.

Understanding the interplay between genetic predisposition and environmental factors is critical to improving cancer treatment outcomes in animals. Genetic mutations, such as those found in the *BRAF* and *KIT* genes, play an important role in tumour development and in the response to therapy. For example, dogs with certain mutations in the *KIT* gene may respond better to targeted therapies that inhibit this pathway [97]. In addition, environmental factors, such as exposure to carcinogens, can influence cancer risk and progression [99]. By integrating genetic testing and understanding environmental influences, veterinarians can develop personalised treatment plans that optimise the use of targeted therapies, improving survival rates and quality of life for affected animals.

### 2.2. Autoimmune and Inflammatory Diseases

A considerable body of evidence has pointed to the fact that autoimmune and inflammatory diseases are among the most common health problems affecting dogs and cats, with a significant impact on their quality of life. Conditions, such as canine atopic dermatitis, rheumatoid arthritis, and systemic lupus erythematosus, are often difficult to manage and treat, highlighting the need for effective treatment strategies tailored to the unique needs of these animals [100,101]. Autoimmune and inflammatory diseases therefore represent a significant health challenge in veterinary medicine, affecting a wide range of species, particularly dogs and cats [100]. Canine atopic dermatitis, rheumatoid arthritis, and systemic lupus erythematosus (SLE) are characterised by inappropriate immune responses, leading to tissue damage and chronic inflammation. In canine atopic dermatitis, for example, the immune system overreacts to environmental allergens, leading to itching, inflammation, and secondary infections [102]. Similarly, rheumatoid arthritis is a debilitating disease involving joint inflammation and pain that severely limits the quality of life of affected animals [103]. Understanding the underlying mechanisms of these diseases is critical to the development of targeted therapies that can effectively manage symptoms and improve overall health.

Canine atopic dermatitis is a common allergic skin disease resulting from an immune system hypersensitivity to environmental allergens [101]. Targeted therapies, particularly Janus kinase (JAK) inhibitors, such as oclacitinib, have emerged as promising treatment options. These inhibitors work by blocking specific cytokine signalling pathways that are central to the inflammatory response associated with allergies. By inhibiting the activity of JAK enzymes, oclacitinib reduces the production of pro-inflammatory cytokines, leading to a significant reduction in itching and inflammation in affected dogs [104,105]. Clinical trials have demonstrated the efficacy of JAK inhibitors in improving the clinical signs of atopic dermatitis, providing a more targeted and less systemic approach than traditional therapies such as corticosteroids [106].

Rheumatoid arthritis in dogs and cats is an autoimmune disease characterised by chronic inflammation of the joints, leading to pain, swelling, and eventually loss of function [107]. A considerable body of evidence has pointed to the fact that targeted therapies for this disease have focused on modulating the immune system through the use of immune checkpoint inhibitors and therapies that block TNF-α. TNF-α is a pro-inflammatory cytokine playing a central role in the inflammatory processes associated with rheumatoid arthritis [108]. By inhibiting TNF-α, these targeted therapies can significantly reduce joint inflammation and the associated pain, thereby improving the quality of life of affected animals [109]. Ongoing research continues to evaluate the safety and efficacy of these treatments, paving the way for more personalised approaches to managing this debilitating disease.

Systemic lupus erythematosus (SLE) is a complex autoimmune disease that can affect multiple organ systems in dogs, resulting in a variety of clinical manifestations. The pathogenesis of SLE involves dysregulation of B and T cell function, resulting in the production of autoantibodies and widespread inflammation [110]. Investigational therapies for SLE focus on selective targeting these immune cells to restore the balance of the immune response. For example, therapies that specifically inhibit B-cell signalling pathways aim to reduce the production of harmful autoantibodies, while agents that target T-cell activation may help to modulate the inflammatory response [111]. A thorough examination of existing studies in the literature reveals that by understanding the complex interactions between these immune cells, researchers hope to develop more effective treatments that relieve the symptoms of SLE while minimising the risk of adverse effects.

#### 2.2.1. Cardiovascular Disease in Animals

Recent findings have shed light on the critical importance of cardiovascular disease, including pulmonary hypertension, which is a major health problem in veterinary medicine, particularly in dogs [112]. Pulmonary hypertension is characterised by increased blood pressure in the pulmonary arteries, resulting in an increased workload on the right side of the heart; if left untreated, it can lead to serious complication, such as right-sided heart failure. This condition can result from a variety of underlying problems, including congenital heart disease, chronic respiratory disease, and other cardiac abnormalities. Recognising the clinical signs, such as exercise intolerance, coughing, and breathlessness, is essential for timely diagnosis and intervention [113].

The pathophysiology of pulmonary hypertension is based on complex mechanisms involving vascular remodelling, endothelial dysfunction, and an imbalance between vasoconstrictors and vasodilators. A critical factor is the increased production of endothelin-1, a potent vasoconstrictor, which contributes to the narrowing of the pulmonary arteries, as demonstrated by researchers [114]. Targeted therapies have been developed to combat this condition, with phosphodiesterase-5 (PDE-5) and inhibitors such as sildenafil emerging as prominent treatment options. These drugs work by inhibiting the breakdown of cyclic guanosine monophosphate (cGMP), a molecule that promotes vasodilation. By increasing cGMP levels, PDE-5 inhibitors effectively reduce blood vessel constriction, leading to lower pressure in the pulmonary arteries [115].

The clinical implications and benefits of targeted therapies are discussed in several papers [116,117,118]. The use of PDE-5 inhibitors in the treatment of pulmonary hypertension has shown promising results in clinical settings, significantly improving the quality of life of affected dogs. Studies suggest that sildenafil may lead to a reduction in pulmonary arterial pressure, improved exercise tolerance, and an overall better prognosis [119]. In addition, the targeted nature of this therapy minimises the systemic side effects commonly associated with broad-spectrum vasodilators. By focusing on the specific pathways involved in vasoconstriction, these drugs offer a more effective and tailored approach to the treatment of pulmonary hypertension in dogs, highlighting the importance of targeted therapies in veterinary cardiology to improve treatment outcomes and the health and well-being of affected animals [120].

#### 2.2.2. Infectious Diseases

Infectious diseases in animals, such as canine parvovirus, feline viral rhinotracheitis, and heartworm disease, highlight the urgent need for effective treatment strategies, including targeted therapies tailored to individual differences in animal physiology and immune responses [121,122,123]. These targeted therapies aim to control specific pathogens while taking into account the unique genetic, immunological, and metabolic factors that can influence treatment outcomes in different species and breeds. For example, in the case of canine parvovirus, researchers are exploring antiviral agents that selectively inhibit viral replication, which may vary in efficacy depending on the dog’s genetic make-up and overall health [124].

Similarly, therapies for feline leukaemia virus (FeLV) are increasingly focusing on modulators of the immune response, with the aim of boosting the natural defences of individual cats based on their specific immune profiles [125]. The approach to treating heartworm disease also highlights the importance of individualised therapy, as certain dogs may have different levels of tolerance to conventional treatments due to their genetic predisposition or coexisting health problems [126]. By understanding individual patient differences and using targeted therapies, veterinarians can optimise treatment efficacy, minimise side effects, and improve overall health outcomes for animals suffering from infectious diseases. This personalised approach not only increases the effectiveness of interventions but also reflects a growing trend towards precision veterinary medicine, which aims to provide tailored care for each animal based on its unique biological characteristics [127].

Feline infectious peritonitis (FIP) is a severe and often fatal disease caused by a coronavirus that can lead to a wide range of clinical manifestations, including effusive (wet) and non-effusive (dry) forms of the disease. The pathogenesis of FIP involves viral replication within macrophages and subsequent immune-mediated damage to various tissues [128]. Recent advances in targeted antiviral therapies, in particular, the protease inhibitor GC376, have shown promising efficacy in combating this infection. By specifically inhibiting the viral proteases required for replication, GC376 disrupts the lifecycle of the coronavirus, resulting in a significant reduction in the viral load [129]. In addition, nucleoside analogues are being investigated for their potential to interfere with viral RNA synthesis, providing a dual approach to effectively target viral infection at multiple stages of its lifecycle [130,131]. These targeted therapies represent a significant advance in the treatment of FIP, transforming what was once a fatal diagnosis into a more manageable condition.

Leishmaniasis, caused by the protozoan parasite *Leishmania*, is a serious threat to dogs’ health, leading to chronic inflammatory responses and various systemic complications. The immune response to *Leishmania* infection is complex, characterised by an imbalance between pro- and anti-inflammatory cytokines, which contributes to the chronic nature of the disease [132,133]. Targeted therapies are being explored to inhibit the specific immune pathways that exacerbate this inflammation, with the aim of restoring immune balance and enhancing the host’s ability to control the parasite. Research into cytokine signalling modulators and immune checkpoint inhibitors holds great promise for improving outcomes in canine leishmaniasis [134,135]. By focusing on the immune mechanisms involved in the disease, these targeted therapies aim to not only reduce the parasite burden but also mitigate the associated inflammatory damage, ultimately leading to better management of this challenging infectious disease in dogs, as shown in various experiments [133,136].

#### 2.2.3. Endocrine Disorders

Advances in targeted therapies for endocrine disorders, such as canine hyperadrenocorticism and insulinoma, highlight the potential of precision medicine in veterinary medicine. By targeting the molecular mechanisms underlying these conditions, veterinarians can develop more effective treatment plans tailored to the individual needs of each patient [127]. Canine hyperadrenocorticism, commonly known as Cushing’s disease, is characterised by the excessive production of cortisol due to overactivity of the adrenal glands. The condition can be caused by various factors, including adrenal tumours or pituitary disorders, which stimulate excessive cortisol secretion. The pathophysiology involves dysregulation of the hypothalamic–pituitary–adrenal (HPA) axis, leading to increased production of the adrenocorticotropic hormone (ACTH). This in turn stimulates the adrenal glands to produce cortisol, resulting in a range of clinical signs, including increased thirst, urination, appetite, and skin changes [137,138]. Trilostane, a competitive inhibitor of the enzyme 3β-hydroxysteroid dehydrogenase, targets the cortisol synthesis pathway by blocking the conversion of pregnenolone to progesterone, ultimately reducing cortisol production. By specifically inhibiting this enzyme, trilostane helps to normalise cortisol levels and alleviate the clinical signs associated with Cushing’s disease, providing a targeted therapeutic approach to the management of this endocrine disorder [139]. The use of trilostane in Cushing’s disease illustrates how the inhibition of specific enzymes can restore hormonal balance, while targeted therapies for insulinoma demonstrate the importance of targeting the growth factors involved in tumour proliferation [140].

Insulinoma, a tumour of insulin-producing cells (β-cells) in the pancreas, is a major endocrine disorder in both ferrets and dogs. These tumours lead to excessive insulin secretion, resulting in hypoglycaemia and a variety of clinical signs, including weakness, seizures, and lethargy. The underlying mechanism involves the unregulated growth of β-cells, often stimulated by growth factors that promote cell proliferation, as shown in research reports [140,141]. Targeted therapies for insulinoma focus on blocking the signalling pathways that contribute to tumour growth and insulin secretion [142]. One promising approach is to inhibit insulin-like growth factor 1 (IGF-1) receptor signalling, which plays a role in cell growth and survival. By targeting these pathways, therapies aim to reduce the activity of insulin-producing tumour cells, thereby controlling insulin levels and alleviating the symptoms associated with hypoglycaemia [143,144].

#### 2.2.4. Neurological Disorders

**Figure 4:**
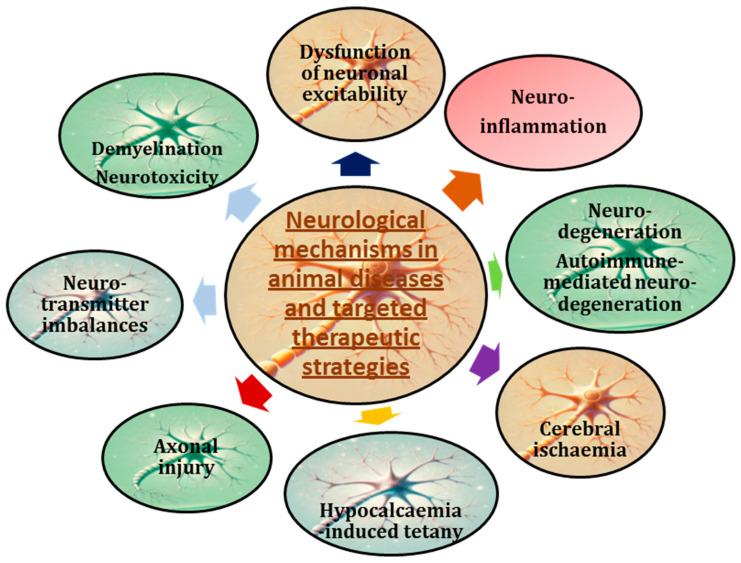
Animal diseases are associated with disturbances in brain function due to factors such as neuroinflammation, oxidative stress and neurotransmitter imbalances. Therapeutic strategies that selectively target these pathways aim to improve treatment outcomes and minimise disease progression [145,146,147,148,149,150,151,152,153,154].

Common neurological disorders in animals include, as follows: canine idiopathic epilepsy [145]; feline hyperesthesia syndrome [146]; degenerative myelopathy [147]; cerebellar hypoplasia [148]; canine cognitive dysfunction [149]; feline leukaemia virus (FeLV)-associated neurological disease [150]; intervertebral disc disease (IVDD) [151]; bacterial meningitis [152]; hypocalcaemic tetany [153]; and chronic inflammatory demyelinating polyneuropathy (CIDP) [154]. The primary goal of targeted therapies for neurological disorders is to address the underlying mechanisms of neurological disease, alleviate clinical symptoms, improve overall quality of life, and promote recovery in affected animals. By focusing on specific pathways and providing personalised treatment plans, veterinarians can optimise outcomes and improve the well-being of their patients (Figure 4).

One study demonstrated that canine idiopathic epilepsy is a common seizure disorder in dogs that is treated with antiepileptic drugs, such as levetiracetam, that stabilise neuronal activity and reduce the frequency of seizures, ultimately improving the quality of life for affected animals [155]. Feline hypersensitivity syndrome is treated with gabapentin to reduce neuronal hyperexcitability, which helps to reduce the abnormal behaviour and discomfort associated with increased sensitivity [156]. There is currently no cure for degenerative myelopathy, a progressive spinal cord disease; however, therapies, such as physical rehabilitation and nutritional supplements, aim to slow the disease progression and improve mobility and quality of life in affected dogs [157].

In cerebellar hypoplasia, supportive care, including physiotherapy, is used to improve coordination and safety in both cats and dogs and to address the challenges posed by this developmental disorder [158]. Finally, canine cognitive dysfunction (CCD), which is similar to Alzheimer’s disease in humans, is treated with drugs, such as selegiline, that improve cognitive function and reduce anxiety in older dogs, targeting both behavioural and cognitive improvements [159].

Findings have confirmed that feline leukaemia virus (FeLV)-associated neurological disease can be treated through the use of antiviral therapies, such as AZT (zidovudine), designed to suppress viral replication and alleviate neurological symptoms in infected cats [160,161]. For intervertebral disc disease (IVDD), surgery combined with anti-inflammatory drugs is used to relieve spinal cord compression, with the aim of relieving pain and restoring mobility [162]. Bacterial meningitis is treated with targeted antibiotic therapy to eradicate the infecting bacteria, with the primary aim of reducing inflammation and preventing permanent neurological damage [163]. The treatment of hypocalcaemic tetany focuses on calcium supplementation to restore normal calcium levels, with the aim of effective reducing the muscle tremors and seizures associated with this condition [164]. Finally, chronic inflammatory demyelinating polyneuropathy is treated with immunosuppressive drugs to reduce the immune response, help the nerves to heal, and improve function and recovery in affected dogs [165].

#### 2.2.5. Common Renal Diseases in Animals

Previous research has shown that chronic kidney disease (CKD), causing a progressive decline in kidney function, is commonly seen in older dogs and cats, leading to symptoms such as increased thirst, urination, and weight loss. Treatment for CKD often involves dietary changes, fluid therapy, and medications to control blood pressure and phosphate levels [166,167]. Acute kidney injury (AKI) refers to a sudden and often reversible decline in kidney function caused by factors such as toxins, infection, or dehydration. Investigations by Ross (2022) and Segev et al. (2024) suggest that therapy for the condition should focus on hydration, treatment of the underlying cause, and supportive care, e.g., diuretics or anti-nausea medications [168,169]. Glomerulonephritis, an inflammation of the kidney’s filtering units (glomeruli), can lead to proteinuria and is often secondary to systemic disease; targeted therapies may include corticosteroids and immunosuppressants to reduce inflammation and manage symptoms [170].

Urinary tract infections (UTIs) can affect kidneys, particularly in cases of pyelonephritis, leading to inflammation and possible kidney damage if left untreated. Appropriate antibiotic therapy is essential to eradicate the infection [171]. Renal tubular acidosis is a disorder in which the kidneys fail to excrete acids into the urine, leading to a build-up of acid in the body. The treatment usually involves bicarbonate supplementation and treatment of any underlying causes [172]. Renal lymphoma is a type of cancer that affects the kidneys, often with signs of kidney dysfunction and systemic disease. The treatment may include chemotherapy, immunotherapy, and supportive care to manage symptoms [173,174]. Polycystic kidney disease is a genetic disorder in certain breeds of cats and dogs, characterised by the formation of cysts in the kidneys. Its management typically focuses on dietary support and regular monitoring, as there is no definitive cure [175].

Nephrolithiasis, or kidney stones, refers to the formation of stones in kidneys that can cause pain, obstruction, and damage to kidney tissue. Treatment options include increased fluid intake, dietary changes, and, if necessary, surgical removal of the stones [176]. Feline idiopathic cystitis primarily affects the bladder but can lead to secondary kidney problems in cats due to urinary obstruction or recurrent infections. Evidence presented by He et al. (2022) indicates that the treatment of the disease may include pain relief, anti-inflammatory medications, and environmental enrichment to reduce stress [177]. Finally, hydronephrosis occurs when a kidney becomes swollen as a result of the accumulation of urine due to a blockage in the urinary tract, which can eventually lead to kidney damage. The treatment involves relieving the blockage, often by surgery, and supportive care to manage kidney function [178].

#### 2.2.6. Ophthalmic Diseases in Animals

Cataracts are a clouding of the lens of the eye, leading to impaired vision or blindness. Its treatment typically involves surgical removal of the cataract and replacement with an artificial lens to restore vision [179]. Glaucoma is characterised by elevated intraocular pressure, which can lead to optic nerve damage and vision loss. Therapies for the condition include medications, such as topical prostaglandin analogues and carbonic anhydrase inhibitors to lower intraocular pressure and, in severe cases, surgical options, e.g., laser procedures or drainage implants [180]. Corneal ulcers are open wounds on the cornea caused by trauma, infection, or underlying disease; their treatment often includes topical antibiotics, anti-inflammatory medications, and surgery, in some cases, to promote healing and prevent complications [181]. Keratoconjunctivitis sicca (dry eye) is a condition in which the eyes do not produce enough tears, leading to irritation and inflammation. Therapies typically include artificial tears and medications, such as cyclosporine A, to stimulate tear production [182]. Progressive retinal atrophy (PRA) is a hereditary condition that causes degeneration of the retina and gradual loss of vision. There is currently no cure and management of PRA focuses on providing a safe environment and support for affected animals [183]. Finally, entropion is a condition where the eyelids roll inwards, causing the eyelashes to irritate the cornea; treatment often involves surgical correction to reposition the eyelids and prevent further irritation or damage to the eye [184].

#### 2.2.7. Bone Disorders in Animals

A thorough examination of the existing literature reveals that osteoarthritis is a degenerative joint disease that leads to the breakdown of cartilage and changes in the underlying bone, causing pain and reduced mobility. Therapies may include non-steroidal anti-inflammatory drugs (NSAIDs), weight management, physical rehabilitation, and joint supplements, such as glucosamine and chondroitin sulphate, to help to manage pain and improve joint function [185]. Osteosarcoma is a malignant bone tumour most commonly found in large-breed dogs; treatment usually involves surgical amputation of the affected limb combined with chemotherapy to reduce the risk of metastasis and prolong survival [186].

Hip dysplasia is a hereditary condition in which the hip joint does not fit properly into the hip socket, leading to arthritis and pain. Research conducted by Dycus et al. (2021) revealed that therapies for the condition include weight management, NSAIDs, physiotherapy, and, in severe cases, surgical options such as hip replacement or femoral head osteotomy to relieve pain and improve mobility [187]. Fractures of the bone can be caused by trauma, stress, or underlying disease. The treatment depends on the type and location of the fracture and may include stabilisation with splints, casts, or surgical fixation with plates or pins to ensure proper healing [188]. Osteomyelitis is an infection of the bone that can result from trauma or surgery; treatment usually consists of a prolonged course of antibiotics, and, in some cases, surgery to remove infected or necrotic bone tissue [189]. Finally, panosteitis is a painful condition characterised by inflammation of the long bones, often seen in young, rapidly growing dogs. The treatment often includes pain management with NSAIDs and supportive care, as the condition is usually self-limiting and resolves with maturity [190].

### 2.3. Epigenetic and Paragenetic Influences on Therapeutic Response

Numerous studies have established that epigenetic and paragenetic influences play a critical role in determining the therapeutic response in animals by affecting gene expression without altering the underlying DNA sequence. Epigenetic modifications, such as DNA methylation and histone acetylation, can influence the expression of genes in response to environmental factors or therapeutic interventions [191]. For example, changes in the epigenetic landscape can enhance or inhibit the expression of genes involved in drug metabolism, immune response, or cell proliferation, ultimately affecting the efficacy and safety of targeted therapies [192]. In veterinary medicine, understanding these modifications allows for the design of personalised treatment strategies that take into account the unique epigenetic profile of individual animals, potentially leading to more effective therapeutic outcomes [191].

Paragenetic factors also play a major role in an animal’s response to therapies, involving interactions between host genetics, the environment, and microbial communities. These factors can alter the expression of genes involved in drug metabolism, immune function, and general health [193]. For example, the gut microbiome can influence the bioavailability of certain drugs and the immune response to infection or disease [194,195]. This interplay between host genetics and environmental influences underscores the complexity of therapeutic responses in animals and suggests that treatments may need to be tailored not only to the disease but also to the animal’s genetic background and lifestyle [196].

Incorporating the knowledge of epigenetic and paragenetic influences into veterinary practice can revolutionise the way therapies are designed and implemented. By identifying specific epigenetic markers or paragenetic factors that correlate with treatment response, veterinarians can develop more personalised approaches to the management of animal diseases [191]. This may involve adjusting dosages, selecting alternative therapies, or implementing lifestyle changes that take into account the animal’s unique genetic make-up and environmental interactions. Ultimately, this integrative approach could increase treatment efficacy, minimise adverse effects, and improve overall animal health outcomes, paving the way for more effective and personalised veterinary care [196].

In summary, it is clear that epigenetic and paragenetic factors play a critical role in determining therapeutic responses in animals. Epigenetic modifications, such as DNA methylation and histone acetylation, influence gene expression and affect the efficacy of targeted therapies, highlighting the need for personalised treatment strategies in veterinary medicine. In addition, paragenetic factors, including interactions between genetics, the environment and the microbiome, further complicate treatment outcomes. It is therefore essential that the veterinary profession develops a deeper and more nuanced understanding of these influences if effective, personalised therapies are to be developed.

### 2.4. Role of Free Radicals and Oxidative Stress in Targeted Therapies

A range of studies provide evidence that free radicals and oxidative stress play an important role in targeted therapies, particularly with their impact on disease progression and treatment efficacy [197]. Free radicals are highly reactive molecules that can damage cellular components such as proteins, lipids, and DNA. They are generated by various metabolic processes, including normal cellular respiration, and can be exacerbated by environmental factors such as pollution, radiation, and toxins [198]. In many diseases, including cancer and inflammatory conditions, an imbalance between free radical production and the body’s antioxidant defences leads to oxidative stress, which can promote disease progression and affect responses to therapy [199]. Targeted therapies often aim to either alleviate oxidative stress or exploit the vulnerability of cells under oxidative stress to increase treatment efficacy [200].

In cancer treatment, the presence of free radicals and the resulting oxidative stress can have both detrimental and beneficial effects. Evidence presented by Aboelella et al. (2021) indicates that tumour cells often have altered redox states, making them more susceptible to oxidative damage than normal cells [201]. Targeted therapies that generate ROS, such as certain chemotherapeutic agents or radiotherapy, can selectively kill cancer cells while sparing healthy tissue. In addition, understanding the oxidative state of the tumour microenvironment can help to refine targeted therapies [202]. For example, combining conventional treatments with antioxidants may protect normal tissues from damage while maintaining therapeutic efficacy against tumour cells that are already under oxidative stress [203].

The role of oxidative stress extends beyond cancer therapy to other areas of veterinary medicine, particularly inflammatory and autoimmune diseases. Free radicals can contribute to chronic inflammation by perpetuating inflammatory pathways, leading to tissue damage and disease progression [204]. In these cases, targeted therapies may aim to reduce oxidative stress through the use of antioxidants or agents that inhibit the production of free radicals. This approach may help to modulate the immune response, reduce tissue damage, and improve overall outcomes in affected animals [205,206].

In summary, the interplay between free radicals, oxidative stress, and targeted therapies is complex and multifaceted. By understanding the effect of oxidative stress on cellular behaviour and therapeutic responses, veterinarians can better tailor treatments for various diseases. This includes exploiting the susceptibility of cancer cells to oxidative damage, reducing oxidative stress in inflammatory conditions, and optimising therapeutic regimens based on the oxidative state of both the disease and the patient [207,208]. Such insights not only improve the efficacy of targeted therapies but also pave the way towards more personalised and effective veterinary care.

### 2.5. Molecular and Physiological Mechanisms of Hypoxia Adaptation as a Model for Targeted Therapy

It is known that animals living in hypoxic environments, such as at high altitudes or in deep-sea habitats, have evolved remarkable molecular and physiological mechanisms to adapt to low oxygen levels; these adaptations can be used as a model for targeted therapies in medicine, as reported by various researchers [209,210,211]. These animals regulate oxygen transport, cellular metabolism, and energy conservation [212,213,214,215] through such mechanisms as the activation of hypoxia-inducible factors (HIFs), which control vital processes, e.g., angiogenesis, red blood cell production, and metabolic adjustments to maintain function in low oxygen conditions [216,217,218]. In human and veterinary medicine, similar strategies are used to treat hypoxia-related conditions, such as ischaemic diseases and cancer, where targeted therapies seek to modulate HIF pathways to improve oxygen delivery and tissue survival. Therefore, understanding these natural adaptations provides crucial insights for the development of gene therapies, pharmacological agents, and other interventions that mimic these responses, offering potential solutions for reducing tissue damage in heart attack, stroke, and even cancer treatment. As such, these evolutionary adaptations in animals provide a promising foundation for the advancement of hypoxia-targeted therapies.

The literature reports the molecular distinctions between animals with high and low tolerances to hypoxia. Animals with high hypoxia tolerance exhibit a greater capacity to stabilise hypoxia-inducible factor-1α (HIF-1α) in low oxygen conditions, which facilitates more effective regulation of gene expression in response to hypoxia. In contrast, animals with low hypoxia tolerance experience faster degradation of HIF-1α, hindering their adaptation to oxygen-limited environments [209,216]. Species with high hypoxia tolerance also display an enhanced regulation of erythropoietin (EPO) production, leading to increased red blood cell formation and improved oxygen transport. Conversely, those with low hypoxia tolerance may have a slower or insufficient EPO response, reducing their ability to counteract oxygen deficiency [217]. High hypoxia-tolerant species show more efficient vascular remodelling through the increased expression of angiogenic factors, such as VEGF, which improves blood flow to hypoxic tissues. Animals with low hypoxia tolerance may have limited or delayed angiogenesis, resulting in poorer oxygen delivery to tissues, as demonstrated by various researchers [201,218].

Animals with high hypoxia tolerance have a more efficient electron transport chain in the mitochondria, based on NADH and FADH_2_. This allows them to optimise ATP production through oxidative phosphorylation, even in low oxygen conditions, by making better use of electrons from NADH and FADH_2_. In contrast, animals with low hypoxia tolerance experience more rapid impairment of the electron transport chain during hypoxia, resulting in reduced ATP production and greater reliance on less efficient anaerobic pathways [212,213]. Additionally, the mitochondria of high hypoxia-tolerant animals are more efficient at sustaining ATP production in low oxygen conditions by utilising alternative pathways, such as glycolysis, more effectively. Meanwhile, animals with low hypoxia tolerance exhibit a greater decline in mitochondrial function, leading to faster energy depletion [212,219,220].

In high hypoxia-tolerant animals, glycolysis-related enzymes, such as PFK (phosphofructokinase) and LDH (lactate dehydrogenase), are upregulated, enabling the animals to maintain energy production through anaerobic metabolism. The body of work in this area has consistently demonstrated that animals with low hypoxia tolerance rely more on oxidative phosphorylation, which is less efficient in low-oxygen environments, as demonstrated in various research publications [210,213,214,219,220]. High hypoxia-tolerant animals have more robust antioxidant defences that reduce the accumulation of damaging ROS in hypoxic conditions. Low hypoxia-tolerant animals tend to accumulate higher levels of ROS, which can lead to oxidative stress and tissue damage [212,213,214,215,216,220,221].

Cells in animals with high hypoxia tolerance demonstrate a stronger resistance to apoptosis in low-oxygen conditions, often through the upregulation of anti-apoptotic proteins like Bcl-2. In contrast, animals with low hypoxia tolerance exhibit higher rates of apoptosis, resulting in greater cellular damage and organ dysfunction [222]. High hypoxia-tolerant animals also exhibit better regulation of ion channels, particularly potassium and calcium channels, which helps to maintain cellular homeostasis and reduces excitotoxicity in the brain and heart. Investigations by Mironova et al. (2010) and Belosludtsev et al. (2020) suggest that animals with low hypoxia tolerance experience ion channel dysregulation, leading to cellular dysfunction during hypoxia [223,224]. Additionally, high hypoxia-tolerant species have increased metabolic flexibility, enabling them to shift between glucose and fatty acid oxidation based on oxygen availability. Those with low hypoxia tolerance are less metabolically flexible, making them more susceptible to energy depletion during extended periods of hypoxia [225,226]. High hypoxia-tolerant animals have more sensitive and rapid oxygen-sensing mechanisms at the tissue level, mediated by proteins such as PHD (prolyl hydroxylase domain) and FIH (factor inhibiting HIF). This allows them to trigger adaptive responses more effectively, whereas animals with low hypoxia tolerance have slower or weaker oxygen-sensing capabilities [227].

In animals with high hypoxia tolerance, the parasympathetic nervous system plays a key role in conserving energy by inducing bradycardia and reducing metabolic demand during hypoxic stress [228]. This mechanism helps to extend the oxygen supply to vital organs. In contrast, animals with low hypoxia tolerance often exhibit a weaker parasympathetic response, resulting in the faster depletion of oxygen reserves. Highly hypoxia-tolerant animals also have a finely tuned sympathetic nervous system that optimises blood flow to critical organs, such as the brain and heart, in hypoxic conditions, while preventing excessive vasoconstriction [219,225,229]. In animals with low hypoxia tolerance, excessive sympathetic activation can lead to harmful effects, such as increased cardiac workload, elevated blood pressure, and higher oxygen demand, worsening the effects of hypoxia. Furthermore, it has been shown that animals with high hypoxia tolerance benefit from coordinated cellular and systemic responses that enhance oxygen utilisation, energy production, and physiological stability during oxygen deprivation [230,231,232].

In summary, the molecular and physiological mechanisms of hypoxia adaptation in animals provide valuable insights for the development of targeted therapies. Animals in hypoxic environments have evolved diverse strategies to regulate oxygen transport and cellular metabolism. These adaptations can serve as models for the treatment of hypoxia-related diseases in human and veterinary medicine, including ischaemic diseases and cancer. Understanding these natural mechanisms will facilitate the development of targeted interventions, such as gene therapies and pharmacological agents, which have the potential to reduce tissue damage and improve outcomes in diseases such as heart attack, stroke, and cancer treatment. This approach highlights the importance of studying hypoxia adaptation as a key model for advancing therapeutic strategies aimed at improving tissue survival and recovery. By harnessing these evolutionary adaptations, researchers can design more precise and effective treatments that mimic the body’s natural response to oxygen deprivation, thereby increasing the effectiveness of therapies aimed at promoting tissue repair and function under low-oxygen conditions. In this way, further research into the differences in hypoxia tolerance between species could lead to tailored therapeutic approaches that take into account the unique physiological responses of each species, contributing to personalised medicine in both veterinary and human care.

### 2.6. Individual Resistance to Hypoxia and Its Implications for Therapy

**Figure 5:**
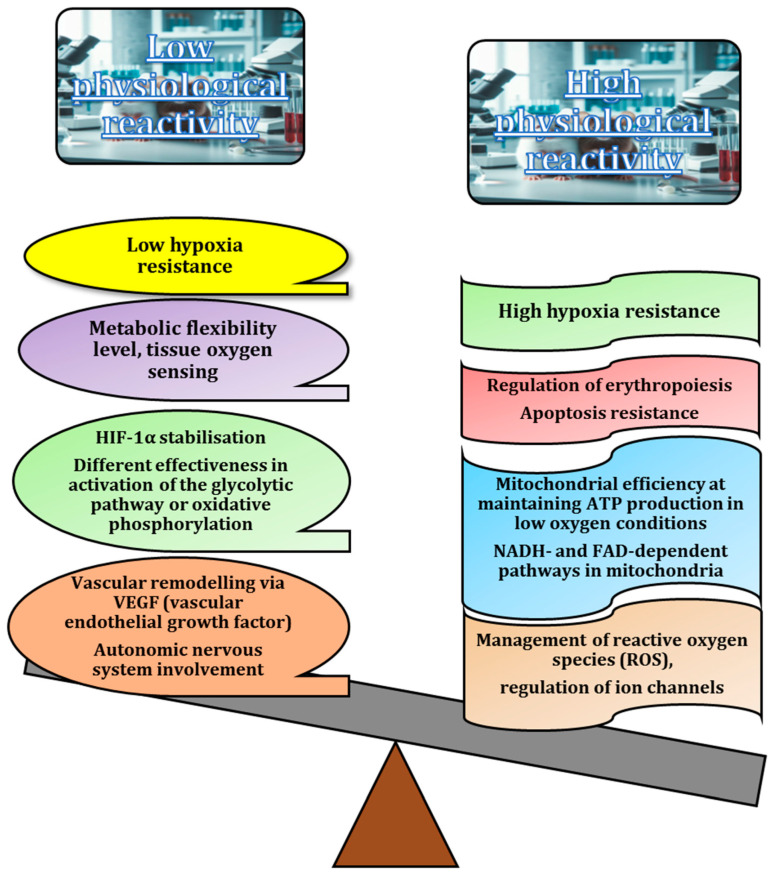
The differences in molecular processes between high and low hypoxia-tolerant animals highlight the critical role of mitochondrial efficiency and physiological regulation in determining the ability of animals to survive and function in hypoxic environments. Highly hypoxia-tolerant animals benefit from coordinated cellular and systemic responses that enhance oxygen utilisation, energy production, and physiological stability during oxygen deprivation [214,215,216,219,222,225].

Many authors have investigated how individual differences in physiological resistance to hypoxia may reflect high levels of autonomic nervous system reactivity, particularly through the balance between parasympathetic and sympathetic activity [210,211,214,232,233]. These systems regulate many vital processes, including the heart rate, blood pressure, and respiratory responses, which are critical in hypoxic conditions. The parasympathetic system generally promotes energy conservation and recovery, while the sympathetic system initiates a stress response to improve oxygen delivery to tissues [234]. This interaction is particularly important in the detoxification or neutralisation of ROS, which increase during hypoxia [235]. ROS are harmful by-products of mitochondrial respiration; excessive production thereof can damage cellular structures. In animals with higher hypoxia resistance, more efficient antioxidant mechanisms, such as superoxide dismutase and glutathione peroxidase activity, help to neutralise ROS and protect tissues from oxidative stress, as shown in many experiments [213,215,220]. This physiological adaptation may explain why certain individuals are better-equipped to tolerate both hypoxic stress and the associated toxic effects of oxidative damage [214,219] (Figure 5).

Although researchers have begun to explore the relationship between individual hypoxia resistance and physiological stress responses, this area remains underexplored, particularly in terms of the different metabolic pathways and their impact on toxicant resistance. Our findings suggest that hypoxia resistance is not simply a reflection of oxygen use, but involves complex biochemical adaptations, including more efficient mitochondrial function and improved ROS management [219]. These adaptations are crucial for understanding how organisms, especially in experimental animal models, cope with stressful conditions such as heavy metal exposure or extreme environments [222,225,228]. Further research is needed to fully understand the metabolic pathways involved and to determine how these mechanisms can be exploited in therapeutic interventions or predictive models of stress tolerance in animals and humans.

It is well known that the efficiency of nitric oxide (NO) storage in animals is genetically determined and closely related to their inherent capacity for NO synthesis, as shown in various studies [219]. Nitric oxide plays a critical role in the regulation of blood flow, oxygen delivery, and cellular metabolism in animals, particularly in stressful conditions such as hypoxia [235]. In animal models, e.g., rodents, the ability to synthesise and store NO varies significantly between individuals, which can affect their overall physiological response to low-oxygen environments [236]. This variation is largely influenced by genetic factors that regulate the production of endothelial nitric oxide synthase (eNOS), which is responsible for NO synthesis [237]. Animals with a higher innate capacity for NO production tend to be more resilient to hypoxia, as they can more efficiently regulate vascular function and tissue oxygen delivery in oxygen-limited conditions [219].

Existing research suggests that different levels of hypoxia resistance are associated with different physiological responses to environmental stressors, including toxicants such as heavy metals, particularly lead [215]. Lead and other heavy metals are known to exacerbate oxidative stress by increasing the production of ROS, which can overwhelm the body’s antioxidant defences. We hypothesised that animals with naturally higher hypoxia resistance would have a superior ability to detoxify various toxicants via their enhanced oxidative stress management systems. In our experiments, we observed that rats with higher hypoxia tolerance had more efficient NO-dependent signalling pathways, which not only helped to maintain vascular tone and blood flow in hypoxic conditions, but also supported detoxification processes. NO has been shown to interact with ROS, reducing their harmful effects and supporting cellular repair mechanisms. This suggests that a robust NO system may be a key factor in the enhanced resilience of hypoxia-sensitive animals to both environmental stresses and metal toxicity [219,228].

Individual resistance to hypoxia in animals is also highly dependent on the functioning of their mitochondrial respiratory chain. Studies conducted by Lukyanova and Kirova (2015) have shown that variations in mitochondrial respiratory efficiency can lead to different levels of oxygen utilisation and energy production in hypoxic conditions [225]. This is related to differences in mitochondrial ion transport and the activity of key mitochondrial enzymes such as cytochrome c oxidase and succinate dehydrogenase [223]. These enzymes are critical for maintaining ATP production during oxygen deprivation, meaning that animals with more efficient mitochondrial function are better-equipped to maintain energy homeostasis during hypoxia. This explains why some animals tolerate hypoxia better than others, a factor that must be considered when using animal models for research or therapeutic interventions involving oxygen deprivation [219,228].

Therapies in animals, especially those targeting conditions associated with oxygen deprivation or hypoxia (e.g., ischaemia or altitude adaptation), must take into account individual hypoxia tolerance. This is because animals with a lower hypoxia tolerance may have different metabolic and physiological responses than those with a higher tolerance [216,219,222]. The activity of the monooxygenase system, as noted by Bayanov and Brunt (1999), also plays a crucial role in determining how animals metabolise drugs and cope with oxidative stress during hypoxia [238]. Inconsistent responses between animals due to variability in NO storage, mitochondrial efficiency, and the biotransformation of xenobiotics can lead to unpredictable outcomes in therapeutic trials. Therapies should therefore be designed with an understanding of individual variations in hypoxia tolerance to ensure that treatment outcomes are both effective and reliable, whilst minimising stress and risk to the animals involved [219,224,226].

In conclusion, it is imperative to recognise the importance of individual hypoxia tolerance in the development of effective therapeutic interventions, particularly in contexts involving stress or heavy metal exposure [215]. Animals with increased hypoxia tolerance have been observed to exhibit enhanced resilience to oxidative stress and toxic insults, highlighting its central role in organismal resilience [214,217,218]. It is therefore essential that therapeutic strategies take this variability into account, with the aim of optimising outcomes by personalising treatments to individual metabolic and physiological profiles, thereby promoting recovery under hypoxic or stress-induced conditions.

Comparative analyses of targeted therapies in different species provide critical insights into how variations in physiology, genetics, and disease pathology influence treatment efficacy. Each species has unique biological characteristics that can affect the absorption, distribution, metabolism, and excretion of drugs, collectively known as pharmacokinetics [239,240]. For example, differences in organ function, body surface area, and metabolic pathways can lead to significant differences in animals’ responses to the same therapeutic agents. Understanding these differences is essential for the development of effective targeted therapies that are both safe and effective for a specific species and highlights the need for tailored treatment protocols in veterinary medicine [239].

In addition, genetic variations between species can significantly affect the expression of drug targets and the mechanisms underlying disease processes. For example, the expression of certain receptors or enzymes involved in drug metabolism may differ between species, affecting the therapeutic outcome of targeted treatments [239,241]. In cancer therapy, certain tumours may express unique species-specific mutations or markers, requiring the development of targeted therapies designed to interact with these specific molecular characteristics. Comparative oncology studies often show that certain therapies that are effective in one species may not translate well to another due to these genetic and molecular differences, highlighting the need for species-specific research and clinical trials [242].

In addition to pharmacokinetic and genetic considerations, the role of the immune system in targeted therapies varies between species. For example, dogs and cats may have different immune responses to immunotherapeutic agents such as monoclonal antibodies or immune checkpoint inhibitors. These differences may be due to differences in immune cell populations, signalling pathways, and the cytokine profiles that modulate therapeutic efficacy [243,244,245]. Understanding how the immune systems of different species interact with targeted therapies can help veterinarians to develop more effective treatment plans and improve patient outcomes by selecting the most appropriate immunotherapeutic strategies for each species [245].

Finally, the comparative analysis of targeted therapies extends to the evaluation of adverse effects and long-term outcomes in different species. Species-specific responses to targeted therapies may affect the safety profile of these treatments and require careful monitoring and management [8]. By studying therapeutic responses and side effects in different species, veterinarians can gain valuable insight into the broader implications of targeted therapies, facilitating the development of guidelines to ensure safe and effective treatment protocols [127,246]. Despite the considerable potential of targeted therapies to advance veterinary medicine, there are several inconsistencies and limitations in the existing literature that require further investigation. For example, the variability in treatment responses between species, and even within the same species, remains poorly understood and is often attributed to genetic, environmental, or epigenetic factors. In addition, the long-term effects and potential adverse outcomes of these therapies are under-reported, particularly in chronic or progressive diseases. The paucity of consistent data on the efficacy of specific molecular targets, as evidenced in some studies, underscores the need for standardised methodologies and broader cross-species investigations. In addition, there is a continuing lack of understanding of the interplay between targeted therapies and the immune system and their role in the management of multifaceted diseases such as cancer or autoimmune disorders. Recognising these limitations requires the implementation of more robust clinical trials, interdisciplinary collaboration, and the integration of advanced molecular techniques to refine therapeutic strategies and ensure consistent results across different animal populations.

## 3. Conclusions

This study is a comprehensive literature review that examines the multiple dimensions of targeted therapies in veterinary medicine. It integrates existing research to highlight the molecular, genetic, and environmental factors that influence treatment efficacy in different species. By exploring these topics, this review highlights the importance of recognising individual species differences, the influence of epigenetic and paragenetic elements, and the role of oxidative stress in shaping therapeutic outcomes. It also discusses the aspects of pharmacokinetics, genetic diversity, and immune responses that are critical to understanding targeted therapies. By bringing these elements together, the review advocates the development of personalised and species-specific treatment strategies with the ultimate aim of improving animal health outcomes.
